# Editorial: Pemphigus and pemphigoid diseases: in memoriam Detlef Zillikens

**DOI:** 10.3389/fimmu.2024.1426834

**Published:** 2024-05-15

**Authors:** Enno Schmidt, Ralf J. Ludwig, Frédéric Caux, Aimee S. Payne, Christian D. Sadik, Takashi Hashimoto, Dedee F. Murrell

**Affiliations:** ^1^ Lübeck Institute of Experimental Dermatology (LIED), University of Lübeck, Lübeck, Germany; ^2^ Department of Dermatology, University of Lübeck, Lübeck, Germany; ^3^ Department of Dermatology, Groupe Hospitalier Paris Seine-Saint-Denis, Bobigny, France; ^4^ Department of Dermatology, Vagelos College of Physicians and Surgeons, Columbia University, New York, NY, United States; ^5^ Department of Dermatology, Graduate School of Medicine, Osaka Metropolitan University, Osaka, Japan; ^6^ Department of Dermatology, St. George Hospital, Kogarah, Sydney, NSW, Australia; ^7^ George Institute of Global Health, Sydney, NSW, Australia; ^8^ Faculty of Medicine, University of NSW, Sydney, NSW, Australia

**Keywords:** autoimmune bullous diseases, pemphigoid, pemphigus, Detlef Zillikens, autoantibody

## Introduction

This Research Topic is dedicated to Detlef Zillikens, MD, director and chair of the Department of Dermatology at the University of Lübeck, Lübeck, Germany, who died in office in September 2022. This article collection in the field of autoimmune blistering diseases (AIBD) was assembled by his long-standing research collaborator colleagues and friends. He intensively worked on AIBD during his entire career and built his department into one of the world’s leading centers for these diseases. With his warm, integrative, open-minded, ever optimistic attitude he was a highly reliable colleague, mentor, and friend to many in the field including each of the editors.

He was a great motivator and an extraordinarily hard-working clinician and researcher who consistently followed his principles and goals. We will never forget his broad knowledge, his passion for research and his patience, his enthusiasm, his empathy, his smiling face and his warm-hearted character. Detlef Zillikens published more than 600 peer-reviewed papers including his most cited review about *Pemphigoid Diseases* in *The Lancet* and the posthumous publication of the identification of laminin β4 as target antigen of anti-p200 pemphigoid, a quest that had occupied him for more than two decades (1, 2).

Several obituaries have already highlighted and valued his scientific work (3–6). Here, within the Research Topic *Autoimmune Blistering Diseases: in memoriam Detlef Zillikens*, the lead article by Hundt et al., details his life and work from different angles and perspectives.

The present Research Topic comprises 36 additional articles on AIBD, many of them referring to and citing the work of Detlef Zillikens. This article collections contains 11 review and opinion articles, 7 case reports and case series, and 18 original research articles.

### Review and opinions on AIBD

Rosi-Schumacher et al. summarize data on the prevalence, incidence, age, and gender of two major AIBD, bullous pemphigoid (BP) and pemphigus vulgaris. An overview about the state-of-the-art diagnosis of AIBD is delivered by (van Beek et al.). Here, clinical features, direct immunofluorescence microscopy and the increasing relevance of detection of serum autoantibodies are described. Huttelmaier et al. provide a systematic literature review about comorbidities in BP following the PRISMA criteria. Of the 48 eligible studies the authors included 34 and calculate associations with neurological and psychiatric diseases, metabolic disorders, and malignancies as well as autoimmune and inflammatory diseases. Patzelt and Schmidt summarize the available data about autoimmunity against laminin 332, a structural protein of the basal membrane zone (BMZ). Laminin 332 is, besides BP180 (type XVII collagen), a major target antigen in mucous membrane pemphigoid (MMP). Repeated injection of anti-laminin 332 IgG in C57BL/6 mice induced major clinical and immunopathological features including fibrosis. Oral lesions were subsequently shown to be ameliorated by dapsone, a first line therapy of MMP (7–9). Of note, following the initial observation by Egan et al. (10) several studies detailed in the present review have corroborated the finding that about a quarter of MMP patients with autoantibodies against laminin 332 have a malignancy. Since a standardized sensitive and specific assay for the detection of serum anti-laminin 332 reactivity is widely available, testing for anti-laminin 332 IgG in all MMP patients is recommended by the European S3 guideline on MMP (11–13). Recently, laminin 332 has also been described in orf-induced pemphigoid (14).

Anti-p200 pemphigoid is characterized by autoantibodies against a 200 kDa protein in the extract of human dermis (15). Subsequently, laminin γ1 and very recently, laminin β4 have been identified as target antigens (2, 16). Gao et al. analyzed the distribution of mucosal lesions, that are present in the majority of patients with anti-p200 pemphigoid, compared to anti-laminin 332 and anti-BP180 MMP. Data about the treatment of pemphigus foliaceus with rituximab and/or high-dose intravenous immunoglobulin are systematically analyzed by Carver et al. They found that both biologics resulted in complete remission in about 63% of patients with a higher relapse rate following rituximab administered by the lymphoma protocol (four times 375 mg/m^2^) compared to two times 1 g.

The so far described mouse models for pemphigus are described in detail by (Emtenani et al.). Furthermore, data about CD4+ T lymphocytes in AIBD, heat shock proteins 90 and 70 in AIBD and COVID, and Janus kinase inhibitors in AIBD are summarized in this Research Topic. Finally, Dmochowski et al. propose a new nomenclature for AIBD incorporating the molecular identity of the target antigens divided into targets being structural proteins or enzymes; the article also highlights pemphigus vulgaris as occurring adjacent to body orifices. The latter were proposed to include atypical immune privileged site as ‘orifices’, including the hair follicles of the scalp, the nipples, and sweat glands in the axillae, genitals, and palms.

### Case reports and case series on AIBD

In a case series of three patients, Foerster al. present the clinical and immunological findings in BP with concomitant HIV-1 infection and give an overview about similar cases in the literature. Three unusual cases of BP in young age, i.e. in a three-month-old boy as well as in association with COVID-19 vaccination in a 43-year old woman and with metastatic colon cancer in a 25-year-old male were described by Rechtien et al.. Corbella-Bagot et al. report on seven BP patients with localized diseases and local triggers such as burns, surgery, rosacea, edema, and paresthesia. A series of 15 BP patients with mucosal involvement not predominating skin lesions and without ocular disease are presented by (Janela et al.). All patients revealed serum IgG against BP180 NC16A with most of them also reacting with C-terminal parts of BP180. Li et al. describe a 55-year-old Chinese woman with multiple myeloma that developed pruritic flaccid blisters, erosions, and pustules on the skin without mucosal involvement. IgA deposits along the dermal-epidermal junction and in an intercellular pattern in the epidermis were seen by direct immunofluorescence of a perilesional biopsy. Unfortunately, the target antigen could not be identified. Furthermore, a case of MMP with multiple autoantibody specificities is reported by Liu et al., and a series of 12 patients with lichen planus pemphigoides with predominant mucosal involvement by Combemale et al.

### Original research on AIBD

In this Research Topic, eighteen original publications are included covering epidemiology, diagnosis, pathophysiology, and treatment of AIBD.

#### Epidemiology and clinical phenotype

While Sollfrank et al. report on a retrospective single-center analysis of AIBD in Middle Franconia, Germany, Kridin et al. identified more than 30 diseases associated with epidermolysis bullosa acquisita based on routine data fueled in the TriNetX database including more than 100 million patients, among them 1,344 patients with latter AIBD. The epidermolysis bullosa acquisita patients showed an increased risk for metabolic and cardiovascular disease, and thrombosis, indicating that these patients should be rigorously investigated and treated to prevent such complications. Lupus erythematosus and lichen planus were the most frequent comorbidities in this entity. Jakowska et al., analyzed involvement of palms and soles that was found in 21 of 462 AIBD patients from Poland, finding no difference in the incidence of these sites between the pemphigus and pemphigoid group. Jukić et al. report HLA class II haplotypes associated in 30 pemphigus vulgaris patients from Croatia compared to 190 controls and showed HLA-DRB1*04:02 not only being highly prevalent in pemphigus vulgaris but also linked to patients with high serum levels of anti-desmoglein 3 IgG.

#### Diagnosis

Schauer et al. compared sensitivity and specificity of indirect immunofluorescence on monkey liver to monkey esophagus in the detection of anti-endomysium IgA employing sera from 16 patients with dermatitis herpetiformis, 67 with coeliac disease, and 20 healthy controls. While sensitivities were comparable in dermatitis herpetiformis, specificity was surprisingly low with monkey esophagus (75%) compared to monkey liver (92%). Höcke et al. reported on a computer-aided classification of indirect immunofluorescence microscopy on primate esophagus and salt-split skin. The established deep network allows the automated recognition of reactivity of AIBD sera on these two substrates differentiating between anti-BMZ and intercellular epithelial binding on esophagus and epidermal and dermal binding on salt-split skin using miniature biochips as previously established for the routine diagnosis of AIBD with manual reading (17–19). This semi-automated algorithm will further facilitate interpretation of indirect immunofluorescence microscopy on these two substrates pivotal for AIBD diagnosis and will also open an avenue for high throughput analyses.

#### Pathophysiology

Schmitt et al. provided an update on the role of pathogenic epitopes within Dsg3 in pemphigus vulgaris using two epitope-specific monoclonal antibodies directed against Dsg3- EC domains EC-1 and EC-5. The data showed that the antibody against EC-5 was less effective in causing loss of cell adhesion, compared to AK23 against EC-1. Both autoantibodies had similar effects on keratin retraction and reduction of desmosome number but only AK23 induced Dsg3 depletion. Moreover, both antibodies induced phosphorylation of p38MAPK and Akt, whereas Src was phosphorylated upon treatment with AK23 only. All pathogenic effects were rescued by p38MAPK inhibition and AK23-mediated effects were also ameliorated by Src inhibition. These data suggest that specific p38MAP kinase inhibitors might be helpful in pemphigus, as was previously proposed. Koga et al. reported 22 patients with BP in whom IL-9 levels were found to be elevated in serum and skin, whereas they were not in patients with epidermolysis bullosa acquisita and normal controls. Four of the patients had serum IL-8 levels measured every 2 weeks; as their BP improved, so did the IL-9 levels, suggesting that IL-9 may play a pathophysiological role. Schinner et al. studied the expression of two transcription factors, T-bet and GATA-3, in skin biopsies from patients with lichen planus, BP, pemphigus vulgaris, and pemphigus foliaceus. They found more GATA-3 in the blistering diseases than lichen planus, whereas all 3 diseases had similar IL-17 signatures. An inhibitor of Fas Ligand, PC111, was tested by Lotti et al. in two human skin organ culture models and in the keratinocyte dissociation assay, showing that this inhibitor could reduce the severity of experimental pemphigus by about 50% and significantly more than normal saline.

Two groups employed the antibody transfer mouse model of epidermolysis bullosa acquisita (20). Akbarzadeh et al. showed that CCR2-deficient mice were significantly less diseased compared to wild type animals and that this effect was not mediated by monocytes, a prominent CCR2-expressing cell type. Seiler et al. demonstrated that mice with neutrophils deficient of C5aR2 were significantly protected confirming a previous study using mice with global deficiency of C5aR2 (21). In an *ex vivo* model of this disease, where incubation of cryosections of normal human skin with anti-type VII collagen IgG and subsequently, with leukocytes from healthy volunteer, induces dermal-epidermal separation (22), Yang et al. screened a natural product library of 800 compounds. Two of them, luteolin peracetate and gossypolone inhibited dermal-epidermal separation in this model.

Finally, Liu et al. investigated the gut microbiome in 66 BP patients compared with the same number of age-, sex-, and study center-matched controls with non-inflammatory skin diseases. They found an overall altered gut microbial community in the BP patients with decreased alpha diversity and reduced *Faecalibacterium prausnitzii* and a greater abundance of pathways related to GABA metabolism. While *F. prausnitzii* is a known microbiomarker for inflammatory diseases, GABA is involved e.g. in inhibition of itch. This study is complementing a multicenter prospective study on the skin microbiome of 228 BP and 190 control patients (23).

#### Treatment

Oren-Shabatai et al. reported the use of rituximab, omalizumab and dupilumab in 9 cases of recalcitrant BP. Rituximab was variably effective in 5/8 patients to get them to a lower dose of corticosteroids. None of these treatments proved very useful in recalcitrant BP but these treatments ought to be assessed in appropriately randomized new onset BP patients. The effect of dupilumab in BP was also investigated by (Yan et al.). In this multicenter retrospective cohort study, 20 BP patients received dupilumab with or without systemic corticosteroids, while 20 BP patients were treated with systemic corticosteroids alone. No differences in the clinical response was noted after 4 and 24 weeks between the dupilumab-treated and the dupilumab-naïve patients. Moreover, levels of serum anti-BP180 IgG did not significantly differ between these two groups, whereas the cumulative corticosteroid dose was significantly lower in the dupilumab arm. When comparing retrospectively the early initiation of corticosteroid-sparing drugs with their late use (after 28 days) in more than 250 BP patients, Fenne et al. did found a lower relapse rate as well as a reduced initial dose of corticosteroids and admission time in the patients with earlier initiation of corticosteroid-sparing medication.

In conclusion, with this Research Topic we would like to honor our mentor and friend, Detlef Zillikens, and will always remember him as kind, integrative, and highly knowledgeable hard-working clinician and researcher who not only left heavy footprints in the field of AIBD but also in our minds and souls.

## Author contributions

ES: Conceptualization, Writing – original draft, Writing – review & editing. RJL: Writing – review & editing. FC: Writing – review & editing. ASP: Writing – review & editing. CDS: Writing – review & editing. TH: Writing – review & editing. DFM: Writing – original draft, Writing – review & editing.
